# Preliminary study on temporal variations in biting activity of *Simulium damnosum s.l*. in Abeokuta North LGA, Ogun State Nigeria

**DOI:** 10.1186/1756-3305-2-55

**Published:** 2009-11-16

**Authors:** Akinola S Oluwole, Uwem F Ekpo, Chiedu F Mafiana, Clement O Adeofun, Olufunmilayo A Idowu

**Affiliations:** 1 Department of Biological Sciences, University of Agriculture, Abeokuta, Nigeria; 2 Academic Planning Unit, National Universities Commission, Abuja, Nigeria; 3 Department of Environmental Management and Toxicology, University of Agriculture, Abeokuta, Nigeria

## Abstract

**Background:**

*Simulum damnosum *Theobald *sensu lato *(*s.l*.) is the vector of the parasitic filarial worm *Onchocerca volvulus *Leuckart which causes onchocerciasis. In order to understand the vector population dynamics, a preliminary 12 months entomological evaluation was carried out at Abeokuta, the Southwest Zone of Nigeria, an onchocerciasis endemic area, where vector control has not been previously initiated. *S.damnosum s.l*. flies were caught on human attractants between 700 to1800 hours each day, for 4 days each month, from August 2007 to July 2008. The flies caught were classified as either forest-dwelling or savanna-dwelling groups based on the colour of certain morphological characters. Climatic data such as rainfall, humidity and temperature were also collected monthly during the period of survey.

**Results:**

A total of 1,139 flies were caught, 596 (52.33%) were forest-dwelling group while 543 (47.67%) were savanna-dwelling group of *S. damnosum s.l*. The highest percentage of forest-dwelling group was caught in the month of August 2007 (78.06%) and the least percentage of forest-dwelling groups was caught in November 2007 (8.14%). The highest percentage of savannah-dwelling group was caught in the month of November 2007 (91.86%) and the least percentage of savannah-dwelling group was caught in August 2007 (21.94%). There was no significant difference between the population of forest and savannah-dwelling groups of the fly when the means of the fly population were compared (*P *= 0.830). Spearman correlation analysis showed a significant relationship between monthly fly population with monthly average rainfall (*r *= 0.550, n = 12, *P *= 0.033), but no significant relationship with monthly average temperature (*r *= 0.291, *n *= 12, *P *= 0.179). There was also a significant relationship between monthly fly population and monthly average relative humidity (*r *= 0.783, *n *= 12 *P *= 0.001). There was no significant correlation between the population of forest-dwelling group of *S. damnosum s.l*. and monthly average rainfall (*r *= 0.466, *n *= 12, *P *= 0.064) and monthly average temperature (*r *= 0.375, n = 12, *P *= 0.115) but there was significant correlation with monthly average relative humidity (*r *= 0.69, *n *= 12, *P *= 0.006). There was significant correlation between savannah-dwelling group and monthly average rainfall (*r *= 0.547, *n *= 12, *P *= 0.033), and monthly average relative humidity (*r *= 0.504, *n *= 12, *P *= 0.047) but there was no significant correlation with monthly average temperature (*r *= 0.142, *n *= 12, *P *= 0.329)

**Conclusion:**

The results from this study showed that both the forest and the savannah dwelling groups of *S. damnosum s.l*. were caught biting in the study area. This could have implications on the transmission and epidemiology of human onchocerciasis if not monitored.

## Background

The *Simulium damnosum *Theobald complex are the only vectors of *Onchocerca volvulus *Leuckart, filarial parasite causing the human onchocerciasis in West Africa [1]. Onchocerciasis disease may be mild (dermatitis) or severe (visual impairment and blindness) and is caused by the human immune response to microfilaria released by female adult worms as they move across subcutaneous tissue and spread throughout the body. Humans are the only known reservoir [2]. The prevalence of human onchocerciasis has been observed to be directly related to the presence and abundance of its vector, *S. damnosum sensu lato (s.l.) *[3]. Early findings have shown that there were geographical variations among West African populations of *S. damnosum s.l*. and that these were related to savannah and forest environment [4-6]. Several studies have shown that the savannah-dwelling vectors of onchocerciasis transmit the blinding form of onchocerciasis while the forest-dwelling vectors transmit the non blinding form of the disease known as onchodermiatiasis [7,8]. Therefore, a reliable identification of biting adult females of *S. damnosum s.l *that transmit the forest and savannah strains of *Onchocerca volvulus *has being considered very essential in the study of transmission and epidemiology of human onchocerciasis [9]. In addition, a detailed understanding of parasite and vector population dynamics is crucial for vector control in the future [10-12]. In Abeokuta, Nigeria, control of onchocerciasis is based on annual mass treatment with Ivemectin (Mectizan^®^) drug, a microfilaricide, as there is no vector control. Therefore, the present work is aimed at identifying *S. damnosum s.l *sibling species biting at Iwofin River in order to provide data on the vector in Abeokuta southwestern zone of Nigeria, which can be useful for vector control of this disease in the future.

## Results

### Population of *S. damnosum s.l*

**Figure 1:**
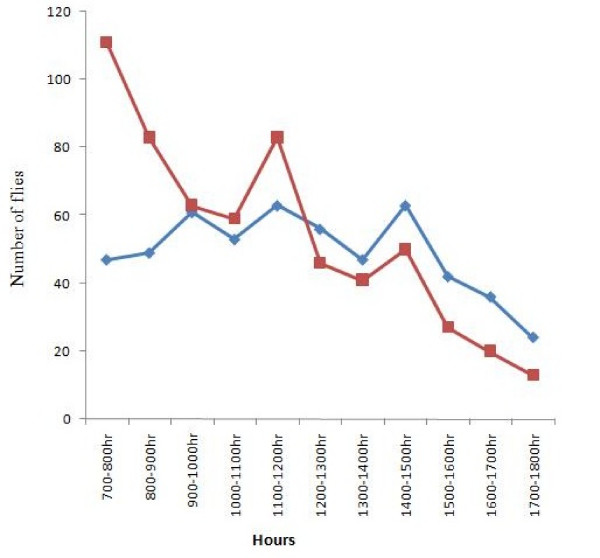
**Biting trend of *S. damnosum s.l*. at Iwofin River, Abeokuta, Southwestern Nigeria**. The red line in the graph represents the biting trend of forest dwelling group, while the blue line represents the biting trend of savannah dwelling group respectively.

Out of 1,139 flies caught at Iwofin River, 596 (52.33%) were identified as forest-dwelling group while 543 (47.67%) were identified as savannah-dwelling group. There was no significant difference between the population of the forest and savannah groups caught when there means was compared (*P *= 0.830). Table 1 shows monthly variation of *S. damnosum s.l*. population with average temperature, relative humidity and average rainfall during the period of study. The populations of the forest-dwelling group were higher than those of the savannah-dwelling group of *S. damno*sum *s.l*. from August 2007 to September 2007, but from October 2007 till June 2008, population of the savanna strains was higher than that of the forest flies. In July 2008, population of forest-dwelling group increased beyond that of the savannah-dwelling group. The highest percentage of forest-dwelling group of *S. damnosum s.l *flies was caught in the month of August 2007 (78.06%) and the least percentage of forest-dwelling groups of *S. damnosum s.l*. flies was caught in November 2007 (8.14%). The highest percentage of savannah-dwelling group of *S. damnosum s.l*. flies was caught in the month of November 2007 (91.86%) and the least percentage of savannah-dwelling groups of *S. damnosum s.l*. flies was caught in August 2007 (21.94%). Figure 1 shows the hourly biting rate of *S. damnosum *in the study area.

**Table 1:** Relative abundance of forest and savannah dwelling groups of *S. damnosum s.l*. caught biting at Abeokuta, southwestern Nigeria from August 2007 to July 2008

Months	Number of flies of savannah dwelling group	Number of flies forest dwelling group	Monthly Total flies	Monthly Average Temperature (°C)	Monthly Average Rainfall (mm)	Monthly Average Relative Humidity (%)
August 2007	52	185	237	31.5	32.3	78.9

September 2007	28	90	119	28.6	16.1	81.5

October 2007	96	45	141	26.2	25.0	76

November 2007	79	7	86	26.7	18.5	65.9

December 2007	46	11	57	25.8	16.4	69.2

January 2008	6	3	9	24.9	0	58.4

February 2008	15	22	37	26.1	0	64.1

March 2008	89	25	114	26.9	24.3	64.2

April 2008	24	11	35	27.3	30.7	63.6

May 2008	28	12	40	27.3	13.8	62.6

June 2008	17	11	28	26.7	23.4	68.8

July 2008	62	174	236	25.6	28.2	73.2

Total/Average	543	596	1139	26.9	19.1	68.9

### Relationship between meteorological data and fly population

The relationship between monthly average rainfall, average temperature, average relative humidity and fly population using spearman non parametric correlation analysis showed that there were significant relationship between average rainfall and fly population (*r *= 0.550, n = 12, *P *= 0.033) and average relative humidity and fly population (*r *= 0.783, *n *= 12 *P *= 0.001) but no significant relationship between average temperature and fly population (*r *= 0.291, *n *= 12, *P *= 0.179). Also there was no significant relationship between forest-dwelling group of *S. damnosum s.l*. with average rainfall (*r *= 0.466, *n *= 12, *P *= 0.064) and average temperature (*r *= 0.375, n = 12, *P *= 0.115) but there was significant relationship with relative humidity(*r *= 0.69, *n *= 12, *P *= 0.006). However, there was significant relationship between savannah-dwelling group and monthly average rainfall (*r *= 0.547, *n *= 12, *P *= 0.033), and monthly average relative humidity (*r *= 0.504, *n *= 12, *P *= 0.047) but there was no significant correlation with monthly average temperature (*r *= 0.142, *n *= 12, *P *= 0.329).

## Discussion

The epidemiology of onchocerciasis is determined by multiple factors such as vector ecology, parasite biology, host immunology, and social factors. The variation in the pathology of onchocerciasis disease from one ecological zone to another has been attributed to the different sibling's species of *S. damnosum s.l *transmitting different strains of *Onchocerca volvulus *Leuckart. Thus *S. damnosum s.l*. has been divided into forest-dwelling and savannah-dwelling group [13,14]. Savannah-dwelling group of *S. damnosum s.l*. are usually found in savanna zone and the forest dwelling group of *S. damnosum s.l*. are usually confined to the forest zone [13]. In view of this, it is expected that only the forest-dwelling group of *S. damnosum s.l*. will be found in the study area since it is located in the forest zone of Nigeria. The presence of the savannah-dwelling group of *S. damnosum s.l*.in this region could be due to the migration of the savannah-dwelling group of *S. damnosum s.l*. flies into the study area from Northern part of Nigeria which is savannah. This may be due to massive deforestation in the study area, for farming, logging and other agricultural activities [15]. Some studies have reported the impact of deforestation on the abundance and distribution of vectors of diseases [16,17]. The implication of the presence of the savannah-dwelling group of *S. damnosum s.l*. is the possible transmission of the blinding form of onchocerciasis which is associated with savannah-dwelling group [18]. Although there was no significant difference in the population of the two groups, it is however important that the population dynamic of *S. damnosum s.l *in this area be monitored continuously for possible establishment and breeding of savannah dwelling group. Therefore the prospecting and identification of larva stages of *S. damnosum s.l*. using cytotaxonomic methods is urgently needed to truly establish the breeding of savannah dwelling group [19]. The establishment of savannah dwelling group in the area will consequently result in changes in transmission, epidemiology and the pathology of the disease [20].

The relationship between monthly rainfall and population of flies caught at Abeokuta, southwest zone of Nigeria was not significant at 0.001 levels; thus increase in rainfall does not necessary result in increase in *S. damnosum s.l*. population. Also the biting rate of *S. damnosum s.l*. flies caught at the study area shows three peak of biting activity and not the usual two peaks reported by many authors on biting activity of *S. damnosum s.l*. These observations differs from the findings of other investigators in the other forest zones of Nigeria where an increase in rainfall significantly affects fly population and show a diurnal biting rate for *S. damnosum *[21-23]. This could possibly be due to climatic changes in the study area as a result deforestation, and possibly global warming. The decline in *S. damnosum s.l*. abundance in the months of April and May 2008 could be due to washing away of the larval stages in Iwofin River as consequence of flooding [23].

More flies were caught in the rainy season (August 2007) than in the dry season (November 2007 to February 2008). This may have resulted from the stimulus of increased oxygen content of the water during rainy season which causes fly to emerge from pupae coupled with increased amounts of nutrients and availability of pre-imaginal sites, all of which enhance pre-imaginal development of *S. damnosum s.l*., which then results in an increase in the adult fly population during the rainy season [24]. The rainy season has been reported to be accompanied by flooding and phytoplankton (detritus, bacteria, diatoms and filamentous algae) blooms [25]. This phytoplankton is the food required by *Simulium *larvae for its development [22]. However, the higher population of savannah-dwelling group of *S. damnosum s.l*. caught during the dry season support the hypothesis that the savannah-dwelling group of *S. damnosum s.l*. flies migrate southward during the dry season in search of breeding sites and blood meal [22]. The strong positive correlation observed between relative humidity and fly abundance is due to the influence of humidity on biting activity of *S. damnosum s.l*. This is similar to other studies where increase in biting activity of *S. damnosum s.l*. is related to increase in relative humidity [26,27].

## Conclusion

The finding from this study shows that, both the forest and savannah dwelling groups of *S. damnosum s.l*. were caught biting in the study area. There is therefore a need for systematic monitoring of the population of *S. damnosum s.l*. in order to establish a possible invasion and breeding of savannah-dwelling group of *S. damnosum s.l*.in this area. Early warning of changes in species composition of *S. damnosum s.l*. is very important in the detection of any trend of change in transmission and epidemiology of onchocerciasis.

## Materials and methods

### Study area

The study was carried out at Iwofin River (7.24936°N, 3.23321°E). Iwofin River is a fast flowing river that begins at Iwofin and empties into Oyan River. It is a breeding site of *S. damnosum s.l *in Abeokuta North Local Government Area of Ogun State located in the southwest zone of Nigeria. The region lies between altitudes 59 m - 62 m. The area is situated in the forest zone of Nigeria. However, the vegetation is that of a derived savannah, consisting of many grasses and few trees due to local deforestation such as land cultivation, pastoral farming and logging.

### Entomological methods

Adult female of *S. damnosum s.l*. flies were caught on human attractants. Two human attractants were stationed alternatively for one hour from 0700 and 1800 hour at a location few meters from the breeding site of *S. damnosum s.l*. on Iwofin River. The Human attractants sat on a bench with their legs exposed. Any fly perching on the exposed parts was caught before it fed by inverting a small glass tube over it. The caps of the tube were then immediately replaced. All tubes containing flies were labeled to indicate time of capture. Each fly was caught with a separate tube. Collections were made on two consecutive days at every fortnight making a total of four days of collection every month from July 2007 to August 2008.

### Identification of *S. damnosum s.l*. using morphological characters

Adult females of *S. damnosum s.l*. flies caught were identified immediately on the field with the aid of field microscope using taxonomic keys [28] that focused on the colour of morphological characters which include wingtuft, forecoxa, arculus, antennae, 9^th ^abdominal segment setae and scutella setae. The flies were classified as a member of either the forest-dwelling group or savannah-dwelling group. A *S. damnosum s.l*. with a pale colour for all the morphological characters mentioned above is classified as a member of the savannah-dwelling group while a fly with a dark colour for any or all of the morphological characters is classified as a forest fly [28]. Flies that were not identified in the field due to poor visibility were preserved in cotton wool soaked in fresh water and transported to the laboratory where they were later identified as described above.

### Climatological data

Monthly average temperature, monthly average rainfall and monthly average relative humidity of the study area were collected to determine its influence on population dynamics of *S. damnosum s.l*. The data were collected every month from the Hydrology department of the Ogun-Osun River Basin Development Authority, Abeokuta.

## Competing interests

The authors declare that they have no competing interests.

## Authors' contributions

EUF and OAS initiated the study, EUF and OAS designed the study. OAS collected the data and EUF, MCF, ACO and IOA supervised the data collection. OAS and EUF did the data analysis and interpretation; OAS and EUF wrote the manuscript. OAS, EUF, MCF, IOA and ACO reviewed the manuscript.

## Ethical Approval

Approval for the study was obtained from the Ogun State Ministry of health and individuals used as human attractant were informed of the consequences of *S. damnosum s.l*. bite and were treated with ivermectin before and after the study.
